# The involvement of homeobox-C 4 in predicting prognosis and unraveling immune landscape across multiple cancers *via* integrated analysis

**DOI:** 10.3389/fgene.2022.1021473

**Published:** 2022-10-05

**Authors:** Junbo Xiao, Ying Li, Yajun Liu, Yiqian Chen, Zixuan He, Shifang Peng, Yani Yin

**Affiliations:** ^1^ Department of Gastroenterology, Xiangya Hospital, Central South University, Changsha, Hunan, China; ^2^ Hunan International Scientific and Technological Cooperation Base of Artificial Intelligence Computer Aided Diagnosis and Treatment for Digestive Disease, Xiangya Hospital, Central South University, Changsha, China; ^3^ National Clinical Research Center for Geriatric Disorders, Xiangya Hospital, Central South University, Changsha, Hunan, China; ^4^ Department of Infectious Diseases, Xiangya Hospital, Central South University, Hunan Key Laboratory of Viral Hepatitis, Changsha, Hunan, China; ^5^ Department of Gastroenterology, Hunan Provincial People’s Hospital, Hunan Normal University, Changsha, Hunan, China; ^6^ University of South China, HengYang Medical School, HengYang, Hunan, China

**Keywords:** HOXC4, pan-cancer, immune modulation, immunotherapy-related analysis, bioinformatic analysis

## Abstract

**Background:** There has been growing evidence that the aberrantly expressed Homeobox-C 4 (HOXC4) plays crucial roles in the development of some cancer types. However, it remains unclear as far as its expression patterns and prognostic significance are concerned, as is tumor immunity.

**Methods:** To investigate the expression levels and prognostic implications of HOXC4, multiple data sources were used in conjunction with quantitative real-time polymerase chain reaction (qRT-PCR) verification. Afterward, diverse immunological-related analyses, along with anti-cancer drug sensitivity, were performed in a number of cancer types. A further exploration of the underlying mechanisms of HOXC4 in tumorigenesis and immunity was carried out using the Gene Set Enrichment Analysis (GSEA) and the Gene Set Variation Analysis (GSVA).

**Results:** Based on extensive database mining, HOXC4 was ubiquitously expressed across 21 tumor cell lines and significantly higher than that of normal tissues in 21 tumor types. The outcome of survival analysis including overall survival (OS), disease-free interval (DFI), disease-specific survival (DSS) and progression-free interval (PFI) revealed that upregulation of HOXC4 expression in several cancers was associated with worse prognosis. Additionally, HOXC4 was observed to correlate closely with colon adenocarcinoma (COAD), head and neck squamous cell carcinoma (HNSC), lower grade glioma (LGG), liver hepatocellular carcinoma (LIHC), rectum adenocarcinoma (READ), and thyroid carcinoma (THCA) in terms of tumor immune cells infiltration. As a result of our comprehensive pan-cancer study, we have identified a significant link between the expression of HOXC4 and the efficacy of immunotherapy-related treatments, together with anti-cancer drug sensitivity. As a final note, HOXC4 was found to modulate multiple signaling pathways involved in tumorigenesis and immunity.

**Conclusion:** HOXC4 has been implicated in our study for the first time as an oncogene in cancers with a poor prognosis, potentially laying the groundwork for promising clinical biomarkers and immunotherapy approaches.

## Introduction

As a worldwide health concern, cancer has gradually imposed a disheartening detriment to society’s well-being and clinical practice (Siegel et al., 2021; Sung et al., 2021). The progress in diagnosing, assessing, and treating cancer continues to advance; however, the disease remains a major financial burden around the world (Bragazzi and Sellami, 2021). As of today, there are not any definite cures. Lack of early diagnosis, local recurrence, distant metastasis, and chemotherapeutic resistance are considered to be the major barriers to poor survival for cancer patients. In light of this, it is imperative that novel methods be explored in order to screen potential diagnostic biomarkers and accordingly to develop corresponding cancer therapy.

Homeobox (HOX) genes, originally associated with developmental process (Gehring and Hiromi, 1986), encode transcription factors that play essential roles for maintaining morphogenesis in multicellular organisms (Arnold et al., 2020). As evidence accumulates over recent decades, dysregulation of HOX genes is implicated in carcinogenesis, the cluster of which Homeobox-C (HOXC) 4 is included (Luo and Farnham, 2020). It has been proved that aberrant expression of HOXC4 contributes to the occurrence and progression of multiple cancers, including prostate cancer, colon cancer, bladder cancer, lung cancer, etc. (Omatu, 1999; Cantile et al., 2003; Leyten et al., 2015). Recently, evidence was presented that HOXC4 can be used to detect prostate cancer at an early stage and predict recurrence, thus indicating its potential as an oncogenic promoter (Miller et al., 2003; Luo and Farnham, 2020). As yet, little is known about the critical role of HOXC4 in pan-cancer.

A great deal of attention has hitherto been paid to immunity-related mechanisms and immunotherapeutics, including determining how immunity interacts with cancer and identifying novel biomarkers for immunotherapy (Gravitz, 2013). It has been established that the tumor microenvironment (TME), especially the tumor immune microenvironment (TIME), is an integral factor of tumor prognosis (Locy et al., 2018). A dramatic shift has occurred from complicated mechanistic protocols to first-line regimens in immunotherapy, which targets microsatellite instability (MSI) and tumor mutational burden (TMB) as well as TME. (Frankel et al., 2017; Chang et al., 2018; Chan et al., 2019; Kalantari Khandani et al., 2020). In recent decades, immunotherapy has shown impressive effectiveness against cancer. There are a number of emerging therapeutic strategies, including PD-1/PD-L1 inhibitors, being used for the treatment of several types of cancer, including colon and lung cancer (Li et al., 2019). However, current checkpoint immunotherapy can only benefit a small number of patients with cancer. To the best of our knowledge, there is still a lack of clarity regarding HOXC4-associated immunotherapy and its underlying mechanisms and functions.

Data for the present study were analyzed from several databases, including the Cancer Genome Atlas (TCGA), Cancer Cell Line Encyclopedia (CCLE), and Genotype Tissue-Expression (GTEx). An in-depth analysis of HOXC4 expression in multiple types of malignancies and its relationship with survival outcomes was performed. In the following analysis, immunological correlations were systematically performed, focusing on HOXC4 expression in different tumor types in relation to TME, immune cell infiltration, MSI, and TMB. Co-expression analysis was then carried out with the mismatch repair (MMR) genes, DNA methylation, and immune-related genes. In addition, Gene Set Enrichment Analysis (GSEA) and Gene Set Variation Analysis (GSVA) were conducted to further explore how HOXC4 might contribute to tumorigenesis. Additionally, various compounds were analyzed in different cell lines to determine the likelihood of resistance to chemotherapy drugs. The findings of this study suggest that HOXC4 may serve as a latent candidate for therapeutic target associated with immunological strategies in a wide variety of cancer types, apart from serving as a prognostic biomarker. HOXC4-associated tumor immunotherapy may yield new insights into personalized treatment and shed new light on HOXC4-associated tumorigenesis.

## Materials and methods

### Data on HOXC4 expression and sample information

Data were obtained from TCGA database on the differential expression of HOXC4, as well as clinical and prognostic outcomes across numerous cancer types (https://portal.gdc.cancer.gov/) (Wang et al., 2016). Data showing insufficient information were excluded (survival time, for example) from a large amount of collected samples. Data from GTEx database was used to analyze HOXC4 expression in 31 normal tissues (https://commonfund.nih.gov/GTEx). HOXC4 gene expression was analyzed in 21 tumor cell lines using the CCLE database (https://portals.broadinstitute.org/ccle/). Our analysis included integration of TCGA and GTEx data to examine the expression differences of HOXC4 between cancer and normal tissues. A log2 (TPM+1) normalization was applied to the whole expression data. This study was conducted according to the flowchart of Supplementary Figure S1 **.**

### Tissue culture, RNA extraction and quantitative real-time polymerase chain reaction

Liver cancer patients with adjacent normal tissue samples (*n* = 8), colon cancer patients with adjacent normal tissue samples (*n* = 4) and breast cancer patients with adjacent normal tissue samples (*n* = 3) were collected from the Pathology Department of Xiangya Hospital, Central South University between December 2021 and March 2022,in which the tissue specimen was obtained in the process of diagnosis and treatment of patients after resection of tumor tissues, and is in full assurance of pathological diagnosis after being apart from the patients, in order to further verify HOXC4 expression (supplementary material of ethic approval).

Briefly, total RNAs (1 ug) were transcribed into cDNAs using the SuperScript III Reverse Transcriptase kit (Invitrogen, Carlsbad, CA, USA). RT-qPCR was performed using Geneseed^®^ qPCR SYBR^®^ Green Master Mix and monitored using the ABI PRISM 7500 Sequence Detection System (Applied Biosystems, Life Technologies). Primers were synthesized by Qiagen (Valencia, CA, USA). GAPDH was used as an endogenous control gene. The RT-qPCR reaction conditions were as follows: hot start at 95°C for 5 min, 40 cycles at 95°C for 10 s, 60°C for 34 s, melting curve stage at 95°C for 15 s, 60°C for 60 s, and 95°C for 15 s. All of the reactions were carried out in triplicate. The experimental data were analyzed using the 2^-△△Ct^ method. The qRT-PCR primer sequences of HOXC4 were as follows: forward: GCC​AGC​AAG​CAA​CCC​ATA​GT, Reverse: CCT​TCT​CCT​TCG​GGT​CAG​GT; GAPDH, forward GGA​GCG​AGA​TCC​CTC​CAA​AAT and reverse GGC​TGT​TGT​CAT​ACT​TCT​CAT​GG. The result was shown in supplementary material of HOCX4 qPCR results.

### Prognosis analysis

Among the aspects of survival analysis, overall survival (OS), disease-specific survival (DSS), disease-free intervals (DFI), and progression-free intervals (PFI) were used to systematically examine the relationship between HOXC4 expression and survival in pan-cancer. Forest plots and Kaplan–Meier curves were used to illustrate the results. By utilizing univariate survival analysis, we calculated the hazard ratio (HR) with 95% confidence interval and log-rank P-value.

### Analysis of HOXC4 expression with TMB, MSI, mismatch repair gene mutation and DNA methyltransferases

The TMB feature in tumor cells promotes immune recognition and correlates with immunotherapy effectiveness (Fusco et al., 2021). An MSI occurs when new alleles are inserted into a tumor as a result of an alteration in microsatellites and is considered one of the hallmarks of immune-checkpoint-related therapy (O'Connell et al., 2020). These scores were computed from somatic mutation data obtained from TCGA. Two radar legends were generated to illustrate the relationship between HOXC4 expression and TMB and MSI, based on Spearman’s rank correlation analysis.

As well, MMR is a process of DNA repair in which unrepairable errors in DNA replication may occur, resulting in a higher incidence of somatic mutations (Armaghany et al., 2012). DNA methylation is a DNA modification mechanism, whose methyltransferases are capable of modulating gene expression and chemical chromatin structure (Szigeti et al., 2018). An evaluation of Pearson correlation analysis between HOXC4 expression levels and mutation levels in five MMR genes (MLH1, MSH2, MSH6, PMS2, and EPCAM) and four methyltransferases’ genes (DNMT1, DNMT2, DNMT3A, and DNMT3B) was conducted.

### Immunological analysis with HOXC4

A database named Tumor Immune Evaluation Resource (TIMER) is intended to provide systematic and integrative data on immune infiltrations in cancer, such as scores for immune cell infiltration. A relationship was estimated between HOXC4 expression and six immune cell infiltration scores, including macrophages, CD8 + T cells, dendritic cells, B cells, CD4 + T cells and neutrophils (Linnebacher and Maletzki, 2012; Yoshihara et al., 2013; Aran et al., 2015; Newman et al., 2015).

Additionally, co-expression analyses were performed on HOXC4 and immune-related genes, including genes encoding MHC, immune activation, immunosuppression, chemokine and chemokine receptor proteins, ferroptosis, m6A and immune checkpoint genes.

In 809 cancer cell lines, GDSC2 datasets (https://www.cancerrxgene.org/) were used to analyze the relationship between HOXC4 expression and half-maximal inhibitory concentration (IC50) values (Yang et al., 2013). To determine IC50 differences of each drug in different gene expression groups, median HOXC4 gene expression was used; then plots concerning IC50 differences and gene expression correlation were generated.

### The biological significance analysis

GSEA and GSVA were performed using normalized RNA-Seq data from TCGA database to examine HOXC4’s underlying functions (Subramanian et al., 2005). As part of the GSEA, GO terms, KEGG pathways, and Reactome data are included. As compared with KEGG analysis, where HOXC4 pathway enrichment was observed, GO analysis concentrated on 3 aspects of regulatory features, namely biology process (BP), cell component (CM) and molecular function (MF). Over 20,000 gene sets are contained in the MSigDB database (version 7.1, updated March 2020; https://www.gsea-msigdb.org/gsea/msigdb/index.jsp), which has been used to determine GSVA scores for cancers (Liberzon et al., 2011). 15 functional pathways were visualized for each tumor showing the most significant correlations with HOXC4 expression.

CancerSEA (http://biocc.hrbmu.edu.cn/CancerSEA/) is the first integrated database that identifies cellular functions at the single-cell level in cancer, covering nearly 41,900 tumor cells, depicting different functional states (such as stemness, invasion, metastasis, proliferation, EMT, angiogenesis, apoptosis, cell cycle, differentiation, DNA damage, DNA repair, hypoxia, inflammation, and quiescence) (Yuan et al., 2019).

### Statistical analysis

Statistic analysis was performed using R software (Version 3.5.3) and GraphPad Prism (version 7.0; GraphPad Software, La Jolla, CA, USA). The Kruskal–Wallis test was used to analyze the differences in HOXC4 expression between tumor cell lines and different normal tissues. For comparing tumor and normal HOXC4 expression, the T-test was used. For correlation analysis, Spearman and Pearson tests were used. It was determined that a statistically significant difference existed at a P-value of <0.05.

## Results

### Pan-cancer with abnormal expression of HOXC4

**FIGURE 1:**
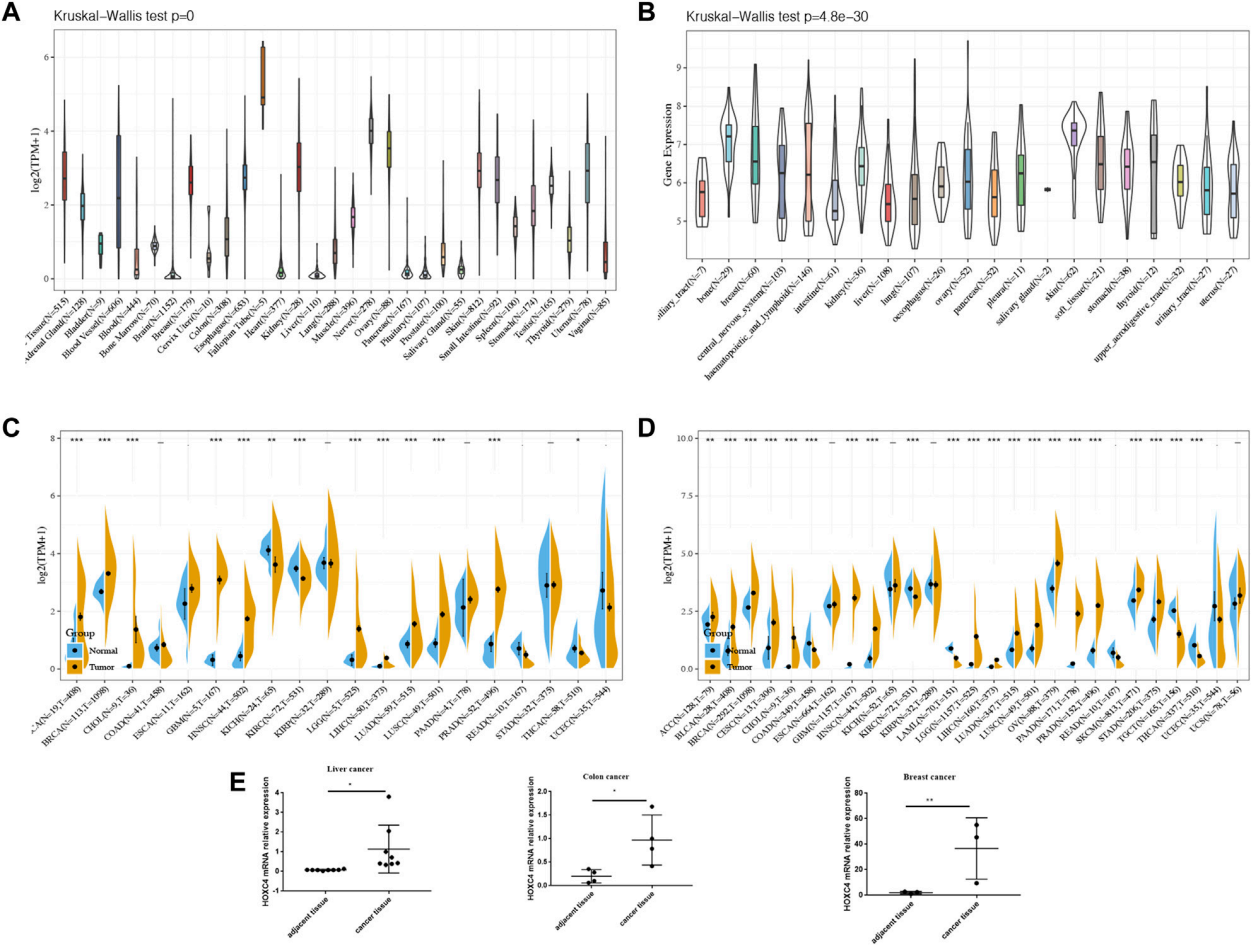
HOXC4 expression in pan-cancer. **(A)** Expression levels of HOXC4 in GTEx dataset. **(B)** Expression levels of HOXC4 the CCLE base. **(C)** Expression levels of HOXC4 in TCGA database. **(D)** HOXC4 expression difference in tumors combined with data of normal tissues I GTEx database and data of TCGA tumor tissues.**(E)** qRT-PCR validation of HOXC4. Cancer vs. adjacent tissue samples from liver cancer, colon cancer and breast cancer patients respectively.*p < 0.05, “*p* < 0.01, *"*p* < 0.001.

Based on the GTEx dataset, Figure 1A shows the pattern of HOXC4 expression in 31 normal tissues. It was found that HOXC4 is extensively expressed in normal tissues, with the fallopian tube showing the highest levels. HOXC4 expression levels were then evaluated according to the CCLE database in Figure 1B, indicating that HOXC4 was expressed ubiquitously across 21 types of tumor cells. As shown in Figure 1C, we compared HOXC4 mRNA levels using the TCGA database to further determine whether HOXC4 was differentially expressed between tumors and normal tissues. A number of cancer tissues, including BLCA, BRCA, CHOL, ESCA, GBM, HNSC, KICH, KIRC, LGG, LIHC, LUAD, LUSC and PRAD, expressed HOXC4 at significantly higher levels than normal tissues. Since TCGA samples of normal tissues were limited, GTEx and TCGA data were then integrated to estimate the HOXC4 expression difference across different cancer types. As a result, higher expression of HOXC4 was observed in 21 tumors, including ACC, BLCA, BRCA, CESC, CHOL, ESCA, GBM, HNSC, KICH, KIRP, LGG, LIHC, LUAD, LUSC, OV, PAAD, PRAD, STAD, TGCT and UCS (Figure 1D). Also, we selected liver cancercolon cancer and breast cancer tissues compared to their adjacent normal ones for qRT-PCR verification due to the limited number of pathological samples available, confirming significant HOXC4 expression upregulation, which was also consistent with our bioinformatic analysis (Figure 1E). Overall, a combined analysis of pan-cancer results reveals that HOXC4 is aberrantly expressed across a variety of cancer types.

### The prognostic significance of HOXC4 expression in various cancer types

**FIGURE 2:**
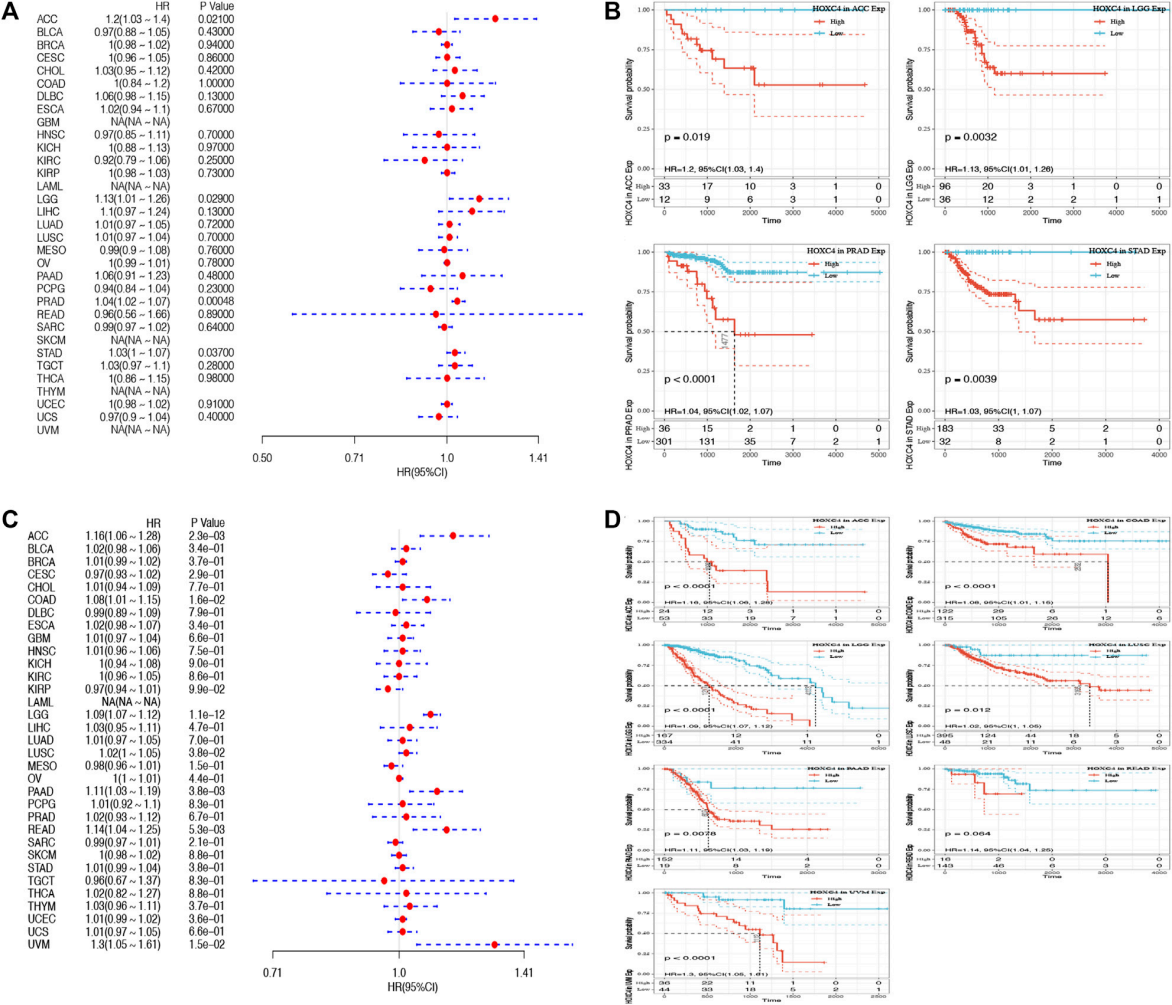
Forest plot and Kaplan-Meier curves of the relationship between HOXC4 expression and DFI and DSS in various types of tumors **(A,B)** HOXC4 expression is associated with DFI analysis in ACC, LGG, PRAD and STAD; **(C,D)** HOXC4 expression is associated with DSS analysis in ACC, COAD, LGG, LUSC, PAAD, READ and UVM.

**FIGURE 3:**
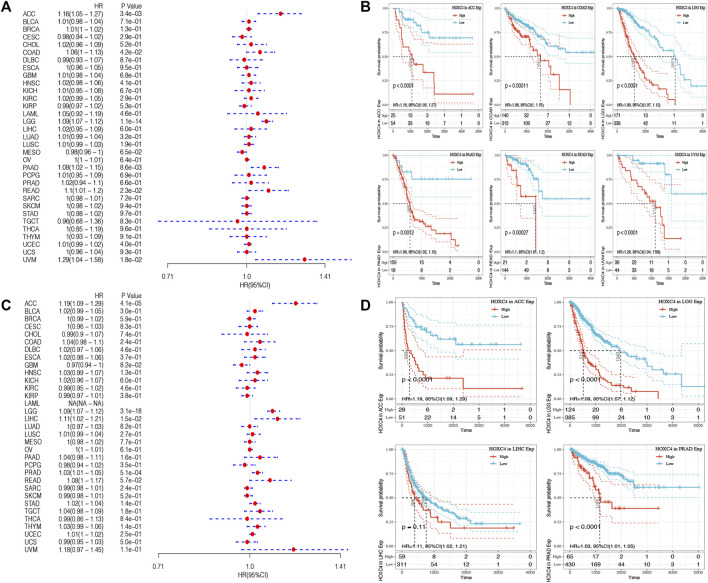
Forest plot and Kaplan-Meier curves of the relationship between HOXC4 expression and OS and PFI in 33 types of tumors **(A,B)** HOXC4 expression is associated with DFI analysis in ACC, COAD, LGG, PAAD, READ and UVM; **(C,D)** HOXC4 expression is associated with PFI analysis in ACC, LGG, LIHC and PRAD.

As yet, no prognostic value has been determined for HOXC4 expression in cancer patients. Thus, Data from TCGA were used to assess the association between HOXC4 expression level and patients’ survival (e.g., DFI, DSS, OS, and PFI). As far as HOXC4 expression is concerned with DFI, univariate survival analysis was conducted, as in Figure 2A. It has been shown that HOXC4 expression is significantly correlated with DFI for patients with four types of cancer, including ACC (*p* = 0.021, HR = 1.2), LGG (*p* = 0.029, HR = 1.13), PRAD (*p* = 0.0048, HR = 1.04) and STAD (*p* = 0.037, HR = 1.03). Additionally, Kaplan-Meier curves comparing DFI for these four tumors (Figure 2B) have shown that patients who express more HOXC4 were more likely to have a worse outcome. In relation to patients’ DSS, HOXC4 expression showed a significant correlation with seven cancer types, including ACC (*p* = 2.3e-03, HR = 1.16), COAD (*p* = 1.6e-02, HR = 1.08), LGG (*p* = 1.1e-12, HR = 1.09), LUSC (*p* = 3.8e-02, HR = 1.02), PAAD (*p* = 3.8e-03, HR = 1.11), READ (*p* = 5.3e-03, HR = 1.14), and UVM (*p* = 1.5e-02, HR = 1.3) in Figure 2C. Kaplan-Meier curves of DSS for these seven tumors also showed increases in HOXC4 expression to be associated with unsatisfactory outcomes (Figure 2D). HOXC4 expression was also associated with patients’ OS in the Forest plot shown in Figure 3A. It was found that HOXC4 expression correlated with patient OS in six cancer types, including ACC (*p* = 3.4e-03, HR = 1.16), COAD (*p* = 4.2e-02, HR = 1.06), LGG (*p* = 1.1e-14, HR = 1.09), PAAD (*p* = 8.6e-03, HR = 1.08), READ (*p* = 2.3e-02, HR = 1.1), and UVM (*p* = 1.8e-02, HR = 1.29). On the other hand, Kaplan–Meier curves comparing OS in these six cancers (Figure 3B) suggested an association between increased HOXC4 expression and a worse prognosis. Further, significant correlation between HOXC4 and patients’ PFI (Figure 3C) was observed in four types of cancer, including ACC (*p* = 1.1e-14, HR = 1.09), LGG (*p* = 3.1e-18, HR = 1.09), LIHC (*p* = 1.5e-2, HR = 1.11), and PRAD (*p* = 5.1e-04, HR = 1.03). Moreover, Kaplan-Meier PFI curves for these four tumors (Figure 3D) showed HOXC4 expression led to a poorer prognosis. Therefore, HOXC4 expression may play an integral role in determining patients’ prognoses across a variety of cancer types.

### Infiltrated immune cells and HOXC4 expression in cancers

**FIGURE 4:**
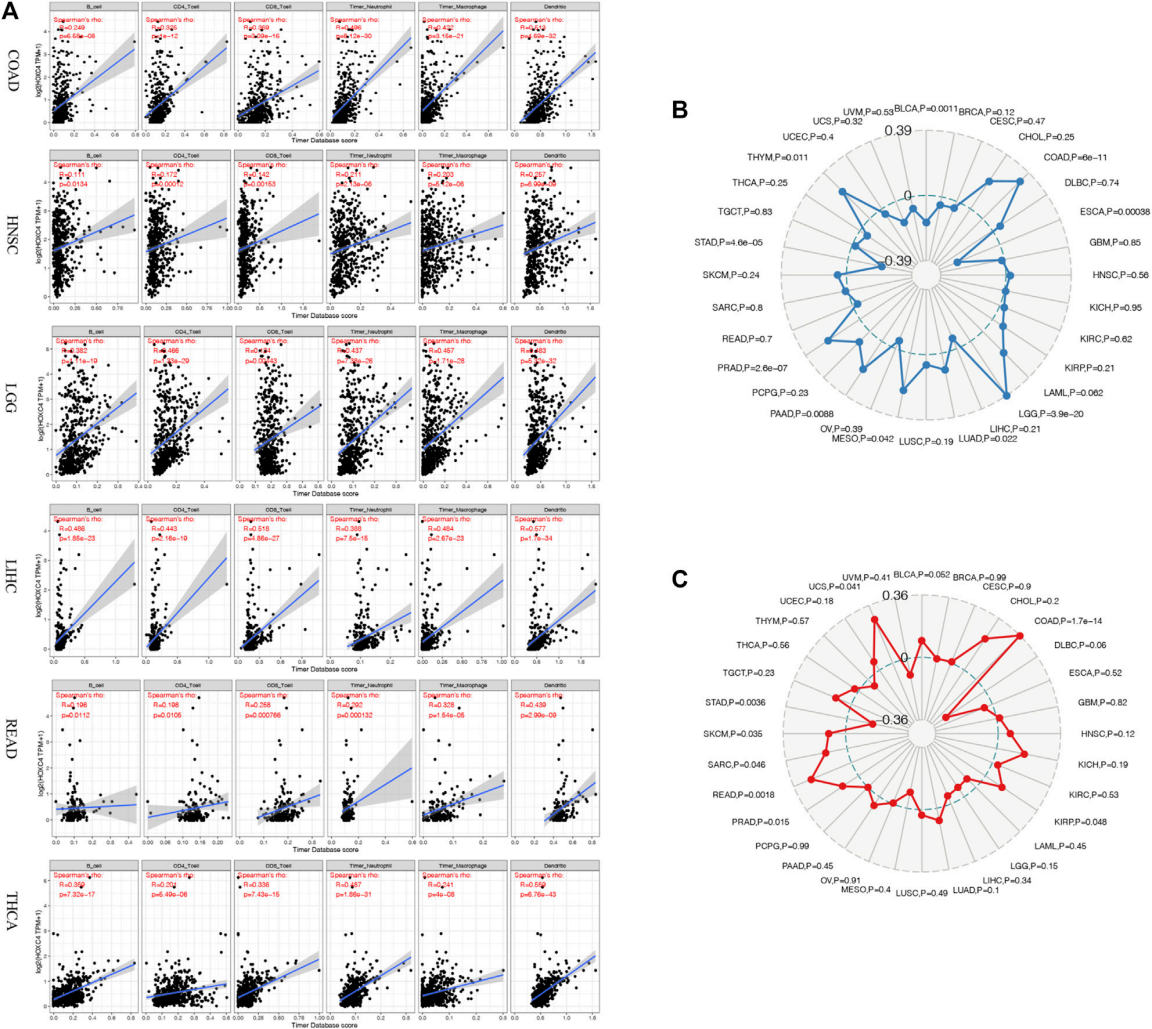
**(A)** Correlation analysis between HOXC4 expression and immune cell infiltration in COAD, HNSC, LGG, LIHC, READ, and THCA. **(B)** Correlation analysis between HOXC4 expression and TMB. **(C)** Correlation analysis between HOXC4 expression and MSI.

TME contains tumor cells and non-tumor elements, the latter of which include stromal and immune cell components (Bolouri, 2015). Although the evaluation of immune cell infiltration has been implicated in various cancers of prognostic significance and cancer-targeted immunotherapy potential, the critical role of HOXC4 expression in TME warrants further investigation. As a result of data provided by TIMER, the relationship between HOXC4 expression and immune cell infiltration was first determined by calculating the score of six immune cells, including B cells, CD4^+^ T cells, CD8^+^ T cells, neutrophils, macrophages, and dendritic cells. Consequently, increased infiltration of macrophages was correlated with an increased expression level of HOXC4 in BLCA, COAD, GBM, HNSC, KIRC, LGG, LIHC, OV, PRAD, READ, SKCM, STAD, TGCT, THCA, and UCEC. As for B cells, CD4^+^ T cells, CD8^+^ T cells, neutrophils, and dendritic cells, their infiltration levels were significantly linked with HOXC4 expression in BLCA, BRAC, CESC, CHOL, COAD, GBM, HNSC, KIRC, KIRP, LGG, LIHC, LUAD, LUSC, MESO, OV, PAAD, PCPG, PRAD, READ, SARC, SKCM, STAD, TGCT, THCA, THYM, UCEC, UCS and UVM. As shown in Figure 4A, infiltration of all six immune cell types exerted significant correlations with HOXC4 expression in COAD, HNSC, LGG, LIHC, READ, and THCA, which provides a solid basis for analyzing immune-related factors. In contrast, no significant correlation was found between immune cell infiltration in ACC, DBLC, KICH, ESCA, or LAML. Detailed information can be found in Supplementary Figure S2. These findings, therefore, suggest that HOXC4 expression was significantly related to immune cell infiltrations and the recruitments of immune cells in various tumor types.

### Expression of HOXC4 with respect to TMB, MSI, MMR gene mutation, DNA methylation, and immune markers

TMB is a marker of genomic alterations that promotes immune recognition, which could induce and accelerate immune recognition associated with the preliminary assessment of immunotherapy response (Sabari et al., 2018). Nonetheless, no relevant reports have been found regarding HOXC4 expression with TMB. BLCA, COAD, ESCA, LGG, LUAD, MESO, PAAD, PRAD, and STAD showed significant correlations between HOXC4 expression and TMB based on Spearman correlation analysis. As illustrated in a radar legend of Figure 4B, its expression in COAD exhibited the strongest correlation with TMB (P = 6e-11).

The MSI was originally seen as a marker of hypermutability in DNA and a potential treatment target for immuno-checkpoint blockade therapy (Hause et al., 2016). Also, few studies have detected the association between HOXC4 and MSI in cancers. We analyzed the relationship between MSI and HOXC4 expression across multiple cancer types using MSI data downloaded from TCGA database. Figure 4C shows that expression of this gene is significantly correlated with MSI in COAD, KIRP, PRAD, READ, SARC, SKCM, STAD, and UCS, with the most significant correlation seen in COAD (*p* = 1.7e-14).

**FIGURE 5:**
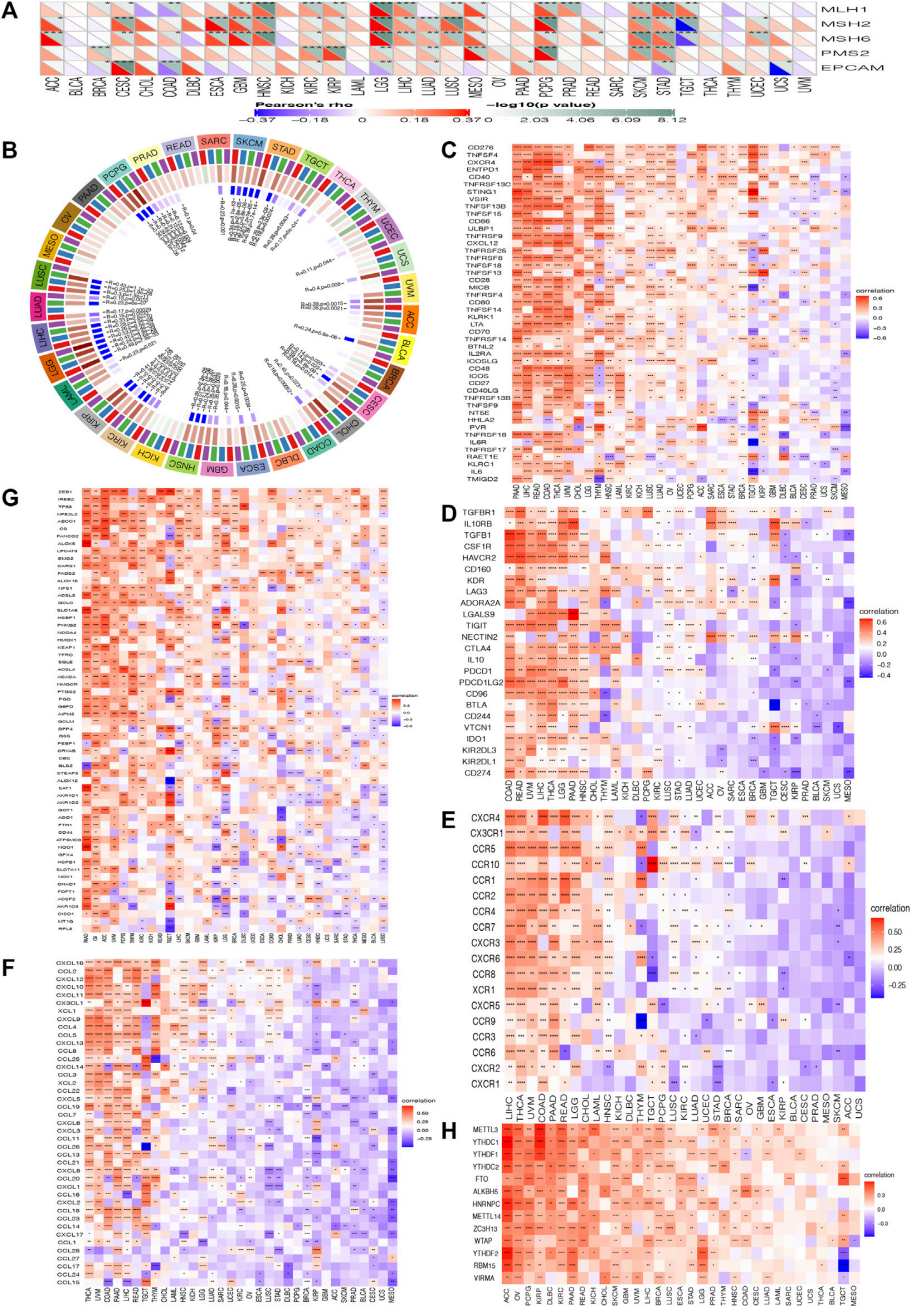
Correlation analysis between the expression of HOXC4 and **(A)** MMR mutation genes **(B)** DNA methyltransferases **(C)** immune activation **(D)** immunosuppressive **(E)** chemokine receptor gene **(F)** chemokine **(G)** ferroptosis **(H)** m6A; ***p < 0.001, **p < 0.01, *p < 0.05, no *p > 0.05.

DNA mismatch errors are repaired by MMR, and failure to correct them can result in more somatic mutations and cancer (McKinney et al., 2020). Therefore, we detected the association between HOXC4 expression and five MMR genes mutation levels (MLH1, MSH2, MSH6, PMS2 and EPCAM). In Figure 5A, HOXC4 expression in ACC, BRCA, CESC, COAD, ESCA, GBM, HNSC, KIRC, KIRP, LGG, LIHC, LUAD, MESO, PAAD, PCPG, PRAD, READ, SKCM, STAD, TGCT, UCEC and UCS correlates with these five MMR genes. As a result, HOXC4 may be able to regulate genes involved in the repair-related genes regarding DNA replication errors to improve survival capability of cancer cells.

Additionally, DNA methylation is another mechanism of methyltransferases that can play a crucial role in DNA modification, the change of which could act as a crucial factor in tumorigenesis (Tiffen et al., 2020). The purpose of this study was to further examine the differential expression of HOXC4 with four DNA methyltransferases, namely DNMT1, DNMT2, DNMT3A and DNMT3B. Figure 5B indicates that HOXC4 expression was significantly correlated with the expression of four DNA methyltransferase genes other than BRCA, DLBC, KICH, MESO, OV, PAAD and THYM, suggesting that HOXC4 may be involved in tumorigenesis of pan-cancer by modifying epigenetic DNA methylation.

**FIGURE 6:**
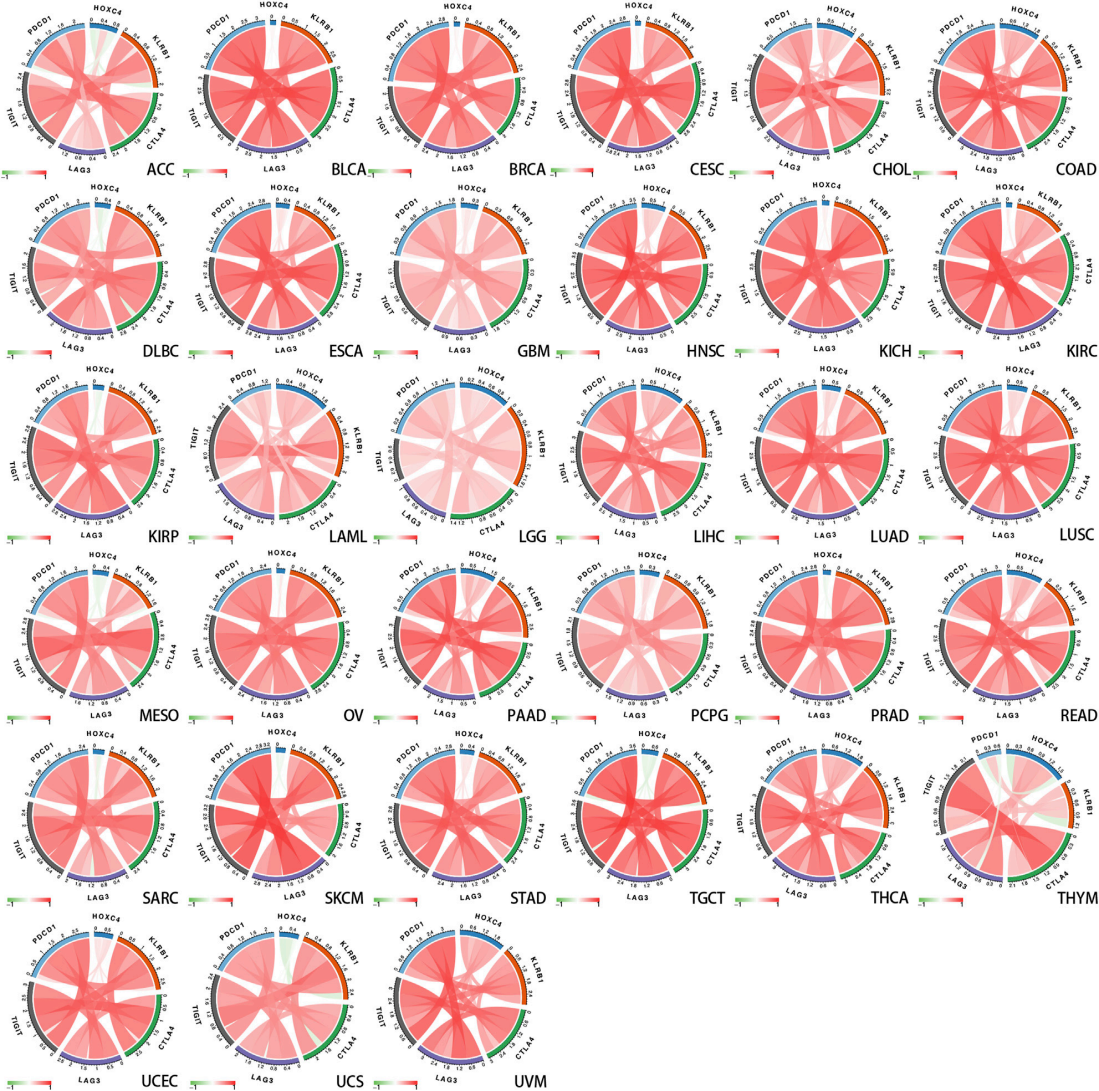
Correlation between HOXC4 expression and five main immune checkpoint genes from TCGA database.

Additionally, it is acknowledged that immune modulation and immune surveillance play vital roles in cancer patients’ prognosis. Co-expression analysis of HOXC4 expression was thus performed with seven immune-related marker gene sets including immune activation, immunosuppression, chemokine receptor proteins, chemokine, ferroptosis, m6A, and immune checkpoint markers. As shown in Figures 5C–F, HOXC4 expression was extensively significantly correlated with immune-activation, immunosuppression, chemokine receptor genes, and chemokine in COAD, HNSC, LGG, LIHC, THCA, etc. Moreover, ferroptosis and m6A gene markers (Figures 5G,H) also exhibited co-expression with HOXC4 in all types of tumors, except UCS. Simultaneous exploration was made concerning the relationship between HOXC4 and five immune checkpoint genes, including LAG3, TIGIT, PDCD1(PD-1), KLRB1 and CTLA4. HOXC4 expression was in close correlation with immune checkpoint genes in BRCA, COAD, HNSC, LGG, LIHC, LUSC, STAD, THYM, etc. (Figure 6). Collectively, the expression level of HOXC4 may be involved significantly in immune regulation and immunological events across pan-cancer.

### GSEA, GSVA and CancerSEA analyses

In view of the significant correlation of HOXC4 expression with immune infiltrations in COAD, HNSC, LGG, LIHC, READ and THCA, GSEA and GSVA analyses were performed to further explore the biological significance of HOXC4 expression in these cancers.

**FIGURE 7:**
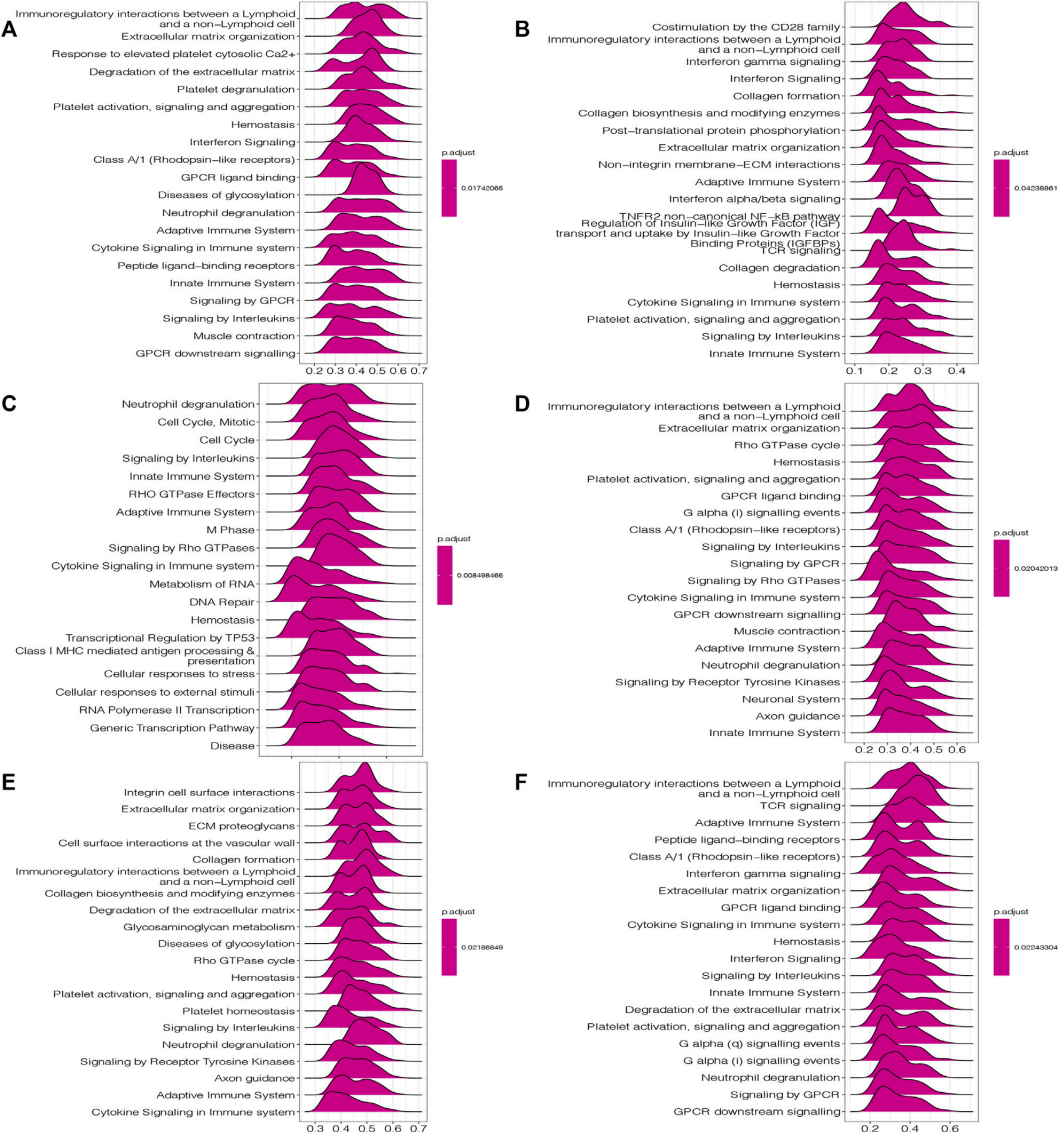
GSEA results of HOXC4 expression involved in Reactome pathways **(A)** COAD; **(B)** HNSC; **(C)** LGG; **(D)** LIHC; **(E)** READ; **(F)** THCA.

GSEA analysis includes GO terms, KEGG pathways and Reactome database. According to Reactome analysis in Figure 7, HOXC4 might be involved in immune-related functions, cell cycle and tumor metabolism in multiple cancers, including the modulation of innate/adaptive immune system, cytokine/interferon/interleukins signaling in the immune system, neutrophil degranulation, B cell receptors, immunoregulatory interactions, class I MHC mediated antigen processing and presentation and several immune-related signaling pathways. As for the potential of HOXC4 in cancer metabolic issues, its expression in LGG, READ, etc. Impacted cellular metabolic process, glycosaminoglycan metabolism, metabolism of RNA, and some metabolic pathways. GSEA results of GO analysis and KEGG pathways was shown in Supplementary Figure S3 **.**

**FIGURE 8:**
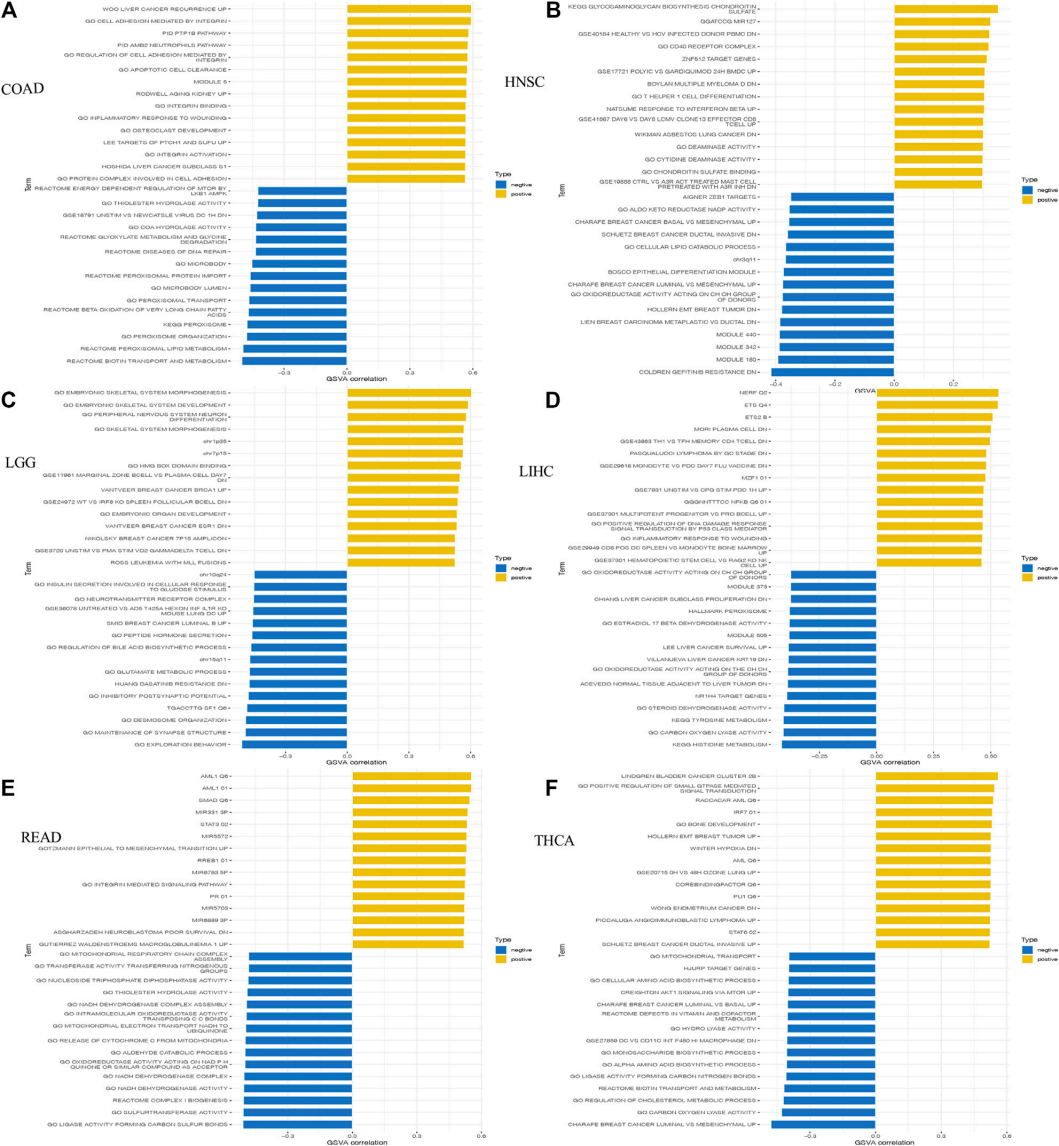
GSVA results of HOXC4 expression involved in **(A)** COAD; **(B)** HNSC; **(C)** I,GG; **(D)** LIHC; **(E)** READ; **(F)** THCA.

GSVA analysis was also conducted to further investigate the underlying biological functions and mechanisms of HOXC4 expression, presented by 15 pathways of the most positive and negative association with HOXC4 in these 6 tumors. The results in Figure 8 illustrated that the expression of HOXC4 was positively related to some immune-related signaling pathways, including B, CD4^+^ T, and CD8 T cells, signal transduction by P53 class mediator, SMAD, STAT3, and PTPIB pathways. In contrast, HOXC4 expression was negatively correlated with cell cycle-related pathways and specific metabolic pathways, such as disease of DNA repair, peroxisomal lipid metabolism, cellular lipid catabolic process, epithelial to mesenchymal transition, etc. Taken these results together, HOXC4 expression may be linked with a variety of biological functions and signaling pathways, especially concerning tumor immunity, cell cycle and metabolic issues.

CancerSEA database contains multitudinous functional states at the cancer single-cell level. To unearth more potential mechanisms, further exploration was performed focusing on the functional state of HOXC4 across 4 types of cancer in CancerSEA database, including brain, skin, breast, and ovary (Supplementary Figure S4). For example, in breast cancer, HOXC4 expression was negatively associated with cell cycle, DNA damage, DNA repair and invasion. Moreover, apoptosis and cell cycle were found to be positively involved in OV, and negatively correlated with DNA repair, hypoxia, proliferation, and stemness, greatly broadening our vision of HOXC4 functional states in cancers.

### Prediction of HOXC4 expression with drug resistance

**FIGURE 9:**
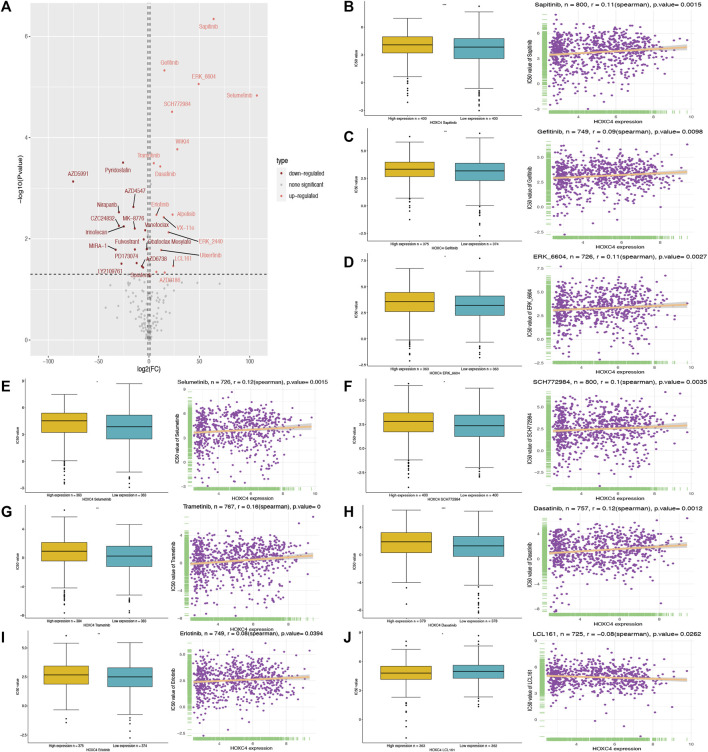
Correlation analysis between HOXC4 expression and anti-cancer drug sensitivity from GSDC2 dataset **(A)** volcano plot visualizes the correlation bewteen high-/low- HOXC4 expression group and different 1,050 values; **(B–J)** 1,050 values of 9 chemotherapy drugs show significant correlation with upregulated expression of HOXC4.

To our knowledge, no study has exploited GDSC data to predict anti-cancer drug sensitivity systematically by comparing the expression of HOXC4 in cancer cell lines and IC50 measurements in drugs. Therefore, Spearman correlation analysis was employed to analyze the association between HOXC4 expression and the IC50 values of 198 drugs in 809 cell lines. Then, we carried out evaluations of drug response prediction in GDSC dataset with their corresponding IC50 values as drug response measurement in comparison between high-/low- HOXC4 expression groups, with corresponding results plotted. As shown in Figure 9, 15 drugs presented significant IC50 differences in high-expression groups, of which only 9 showed a significant correlation between HOXC4 and IC50, including Sapitinib, Gefitinib, ERK_6604, Selumetinib, SCH772984, Trametinib, Dasatinib, Erlotinib, and LCL161. Accordingly, the positive correlation of higher HOXC4 expression with IC50 values may indicate that elevated HOXC4 expression may result in anti-cancer drug resistance and lower chemosensitivity.

## Discussion

Identifications of critical biomarkers with involvement in tumor initiation and progression have sparked great interest in tumorigenesis research. An integrative pan-cancer study of a single gene is by far endowed with significant implications towards early diagnosis, therapeutic regimen and cancer prevention (Qureshi et al., 2020; Srivastava and Hanash, 2020). In the meantime, comprehensive and systematic studies have been undertaken concerning tumor immunity and its influence on immunotherapy (Jiang et al., 2020; Passaro et al., 2020). According to our previous description, HOXC4 plays a vital role during embryonic development and in the development of cancer. However, HOXC4 is not well understood as a molecular biomarker in pan-cancer, nor its expression level. Here, our study uncovers new insights into how aberrant HOXC4 expression plays an important role in tumor immunology and as a potential biomarker for malignancy development.

Based on a comprehensive analysis of the expression of HOXC4 in pan-cancer, the present study found that HOXC4 was widely expressed in 31 normal tissues as well as 21 tumor cell lines, with the highest expression of HOXC4 occurring in the fallopian tube and ovary, respectively. In the meantime, the combined TCGA and GTEx databases revealed abnormal overexpression of HOXC4 in 21 types of cancer, including ACC, BLCA, BRCA, CESC, CHOL, ESCA, GBM, HNSC, KICH, KIRP, LGG, LIHC, LUAD, LUSC, OV, PAAD, PRAD, STAD, TGCT and UCS, which suggests that HOXC4 may function in carcinogenesis as an oncogene. Moreover, a clarification of the relationship between HOXC4 expression and survival outcomes revealed that higher levels of HOXC4 were associated with suboptimal survival outcomes, regardless of OS, DSS, DFI and PFI. Thus, abnormally high levels of HOXC4 expression appear to be crucial for multiple cancers to form and develop.

The TME, which includes the tumor immune microenvironment (TIME), in which immune cells infiltrate, is considered to be one of the “seventh marker feature” of a tumor, comprising an indispensable component of the immune response and tumor progression, along with assessing therapeutic effectiveness and survival outcomes (Junttila and de Sauvage, 2013). There is still uncertainty, however, as to whether the expression of HOXC4 is pivotal to TME. In our study, we observed a significant correlation between HOXC4 expression and 6 immune infiltrating cells: B cells, CD4^+^ T cells, CD8^+^ T cells, dendritic cells, macrophages, and neutrophils in COAD, HNSC, LGG, LIHC, READ, and THCA(41). Several studies have found that dendritic cells and macrophages play an important role in antitumor immunity, which can lead to detrimental tumor immunity escape in the presence of excessive chemotactic factors (Ohtani, 2007). In this manner, infiltration of these immune cells may contribute to impaired immunity and may be closely related to cancers of the six types. Hence, infiltrating immune cells can be regulated by HOXC4 expression during different types of cancer development and immune escape can be influenced.

Previously, we have discussed the significant role that TMB and MSI play in immunotherapy (Gajewski and Schumacher, 2013; Goodman et al., 2017). This study indicates that HOXC4 expression was strongly correlated with TMB in BLCA, COAD, ESCA, LGG, LUAD, MESO, PAAD, PRAD, and STAD, suggesting that HOXC4 is probably responsible for mutation-driven tumorigenesis. Additionally, studies suggest that immunotherapeutic outcomes may be improved with HOXC4 expression in tumors with higher TMB, suggesting that HOXC4 expression may be warranted in tumors with higher TMB. For MSI, significant associations with HOXC4 expression were found in COAD, KIRP, PRAD, READ, SARC, SKCM, STAD, and UCS, which indicates that HOXC4 may serve as a hall marker for patients undergoing immune-checkpoint-blockade therapy. Furthermore, HOXC4 can play a crucial role in determining the oncogenic validity of immune cells and immune-related genes in various cancers, particularly through their co-expression analyses. A mutation in MMR genes or an alteration in DNA methylation markers can lead to a cumulative increase in genetic or epigenetic errors, which can contribute to tumor occurrence (Butler et al., 2020; Georgakopoulos-Soares et al., 2020). A significant correlation was also found between the expression of HOXC4 and the MMR mutation genes in this study, with the exception of BLCA, CHOL, DLBC, KICH, LAML, OV, SARC, THCA, THYM and UVM. Aside from BRCA, DLBC, KICH, MESO, OV, PAAD, and THYM, HOXC4 expression is significantly associated with DNA methyltransferase genes expression. In turn, these results substantiate and support our previous findings. It was further clarified that HOXC4 expression is correlated with immune-related genes, particularly immunosuppressive genes such as PD-1 and PD-L1. As a result of their strongly positive correlation, it is likely that HOXC4 regulates the tumor immunosuppressive microenvironment and functions as a novel target for immunotherapy against related tumors. In conclusion, HOXC4 expression is intimately correlated with tumorigenesis and genes involved in immunity in pan-cancer, supporting the importance of HOXC4 in immune modulation and immunotherapy.

It is well known that ineffective chemotherapy can increase mortality and decrease quality of life in cancer patients. Personalized chemotherapy continues to be a challenging endeavor (Chen et al., 2013; Kim et al., 2016). In our study, to test the significance of HOXC4 expression in chemotherapeutic drug application, tumor cell lines with similar responses to a drug were simulated in a manner similar to that of tumor patients. To date, GDSC is the largest project that evaluates anticancer drug sensitivity and identifies biomarkers for drug response in cancer cell lines. Among the drugs examined in our study, Sapitinib, Gefitinib, ERK_6604, Selumetinib, SCH772984, Trametinib, Dasatinib, Erlotinib, and LCL161 showed significant correlations between upregulated HOXC4 expression and their corresponding IC50 values. Hence, by assessing different anti-cancer drug responses in various patients based on their HOXC4 expression levels, we may eventually be able to improve our individual therapeutic treatment.

Last but not least, we investigated the biological significance of HOXC4 expression. In this study it was discovered that HOXC4 could play a role in cancer pathogenesis through its involvement in a variety of immunological pathways, including *via* immune response, PD-L1 expression and PD-1 checkpoint pathway in cancers, PI3K-AKT signaling pathway, NK-κB signaling, and several metabolic pathways, consistent with previously published studies (Rouce et al., 2016; Yang et al., 2017; Pham et al., 2020). Collectively, HOXC4 plays an important oncogenic role in the development and progression of cancers, as well as in the regulation of these signaling pathways.

In summary, we found that HOXC4 expression differs significantly among tissue types, and that overexpression of HOXC4 is significantly associated with poorer clinical outcomes in pan-cancer. Furthermore, our findings suggest that HOXC4 strongly correlates with TME, including an increase in the level of infiltration of six immune cells across a variety of cancers. It is also noteworthy that the expression of HOXC4 is strongly related to the expression of TMB, MSI, MMR mutation genes, DNA methyltransferases, immune-related markers, and immune checkpoint markers across a broad spectrum of cancer types, all of which affect immunotherapy-related treatment. Further, differential chemosensitivity responses of different cancers could be reflected by upregulation of HOXC4 expression, which could facilitate better tailoring of anticancer therapies. Nevertheless, our results mostly relied on comprehensive and systematic data analysis. We will conduct further basic experimental verification in our subsequent research and HOXC4’s biological activity in different cancer cells will then be explored, such as proliferation and/or migration. Also, we did not analyze the association between HOXC4 expression and immunotherapy cohort, which could be potential indicators of patients’ immunotherapy response. So, the predictive value of HOXC4 regarding the immunotherapy response remains to be well-documented in the future. We hope that our research will provide novel insights into precision medicine for more individualized immunotherapy advancement in the future by elucidating the multifaceted roles HOXC4 plays in tumorigenesis and tumor immunity.

## Data Availability

The original contributions presented in the study are included in the article/Supplementary Material, further inquiries can be directed to the corresponding authors.

## Glossary

ACC: Adrenocortical carcinoma
BLCA: Bladder urothelial carcinoma
BRCA: Breast invasive carcinoma
CESC: Cervical squamous cell carcinoma and endocervical adenocarcinoma
CHOL: Cholangiocarcinoma
COAD: Colon adenocarcinoma
DLBC: Lymphoid neoplasm diffuse large B-cell lymphoma
ESCA: Esophageal carcinoma
GBM: Glioblastoma multiforme
HNSC: Head and neck squamous cell carcinoma
KICH: Kidney chromophobe
KIRC: Kidney renal clear cell carcinoma
KIRP: Kidney renal papillary cell carcinoma
LAML: Acute myeloid leukemia
LGG: Brain lower grade glioma
LIHC: Liver hepatocellular carcinoma
LUAD: Lung adenocarcinoma
LUSC: Lung squamous cell carcinoma
MESO: Mesothelioma
OV: Ovarian serous cystadenocarcinoma
PAAD: Pancreatic adenocarcinoma
PCPG: Pheochromocytoma and paraganglioma
PRAD: Prostate adenocarcinoma
READ: Rectum adenocarcinoma
SARC: Sarcoma
SKCM: Skin cutaneous melanoma
STAD: Stomach adenocarcinoma
TGCT: Testicular germ cell tumors
THCA: Thyroid carcinoma
THYM: Thymoma
UCEC: Uterine corpus endometrial carcinoma
UVM: Uveal melanoma
