# Supplementary figures and images for: The involvement of homeobox-C 4 in predicting prognosis and unraveling immune landscape across multiple cancers via integrated analysis
Source: Front Genet. 2022 Oct 5;13:1021473. doi: 10.3389/fgene.2022.1021473 (PMC9581313; doi:10.3389/fgene.2022.1021473)

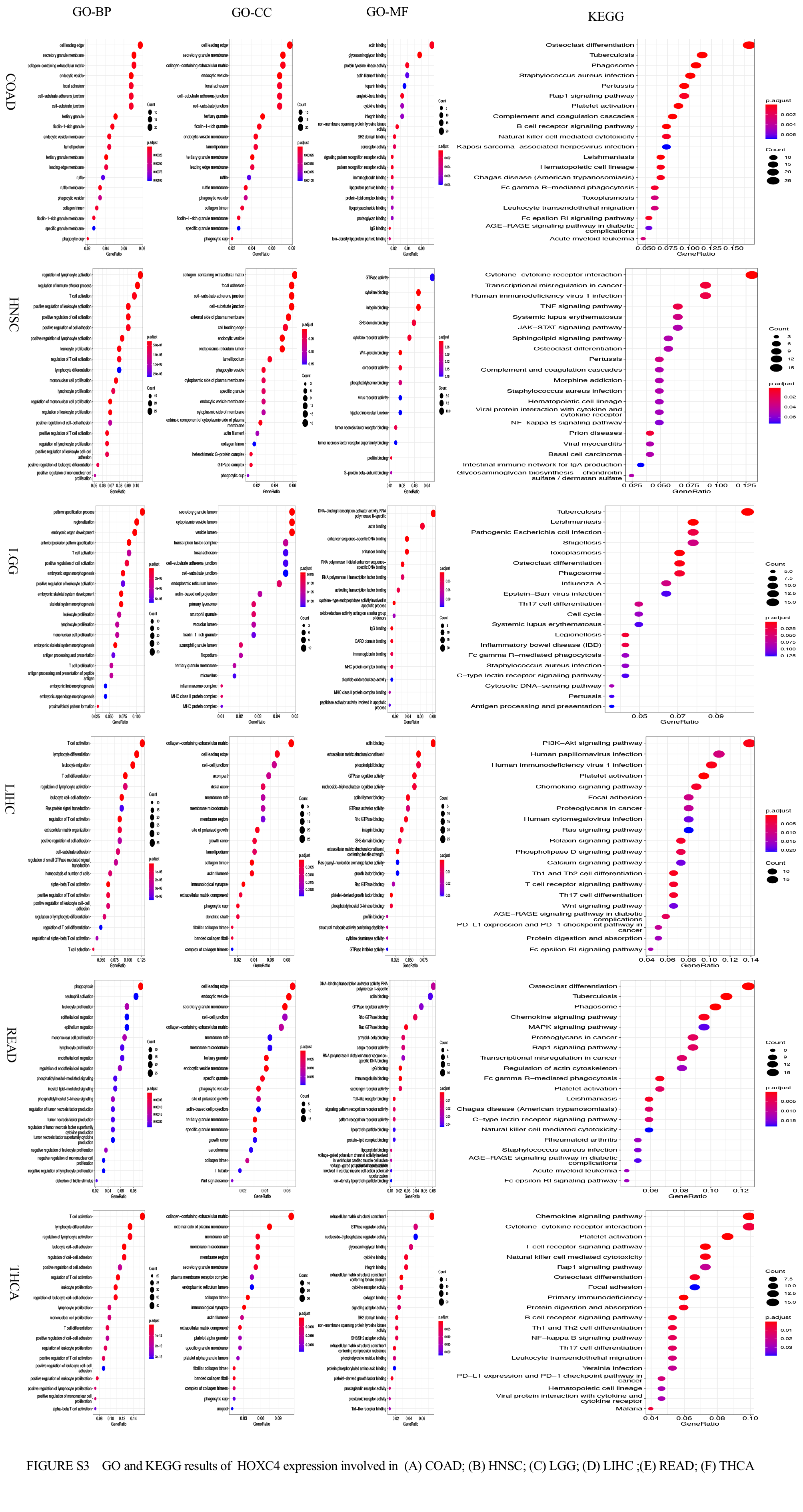

Supplement: Supplementary file 2 [file Image3.TIF]

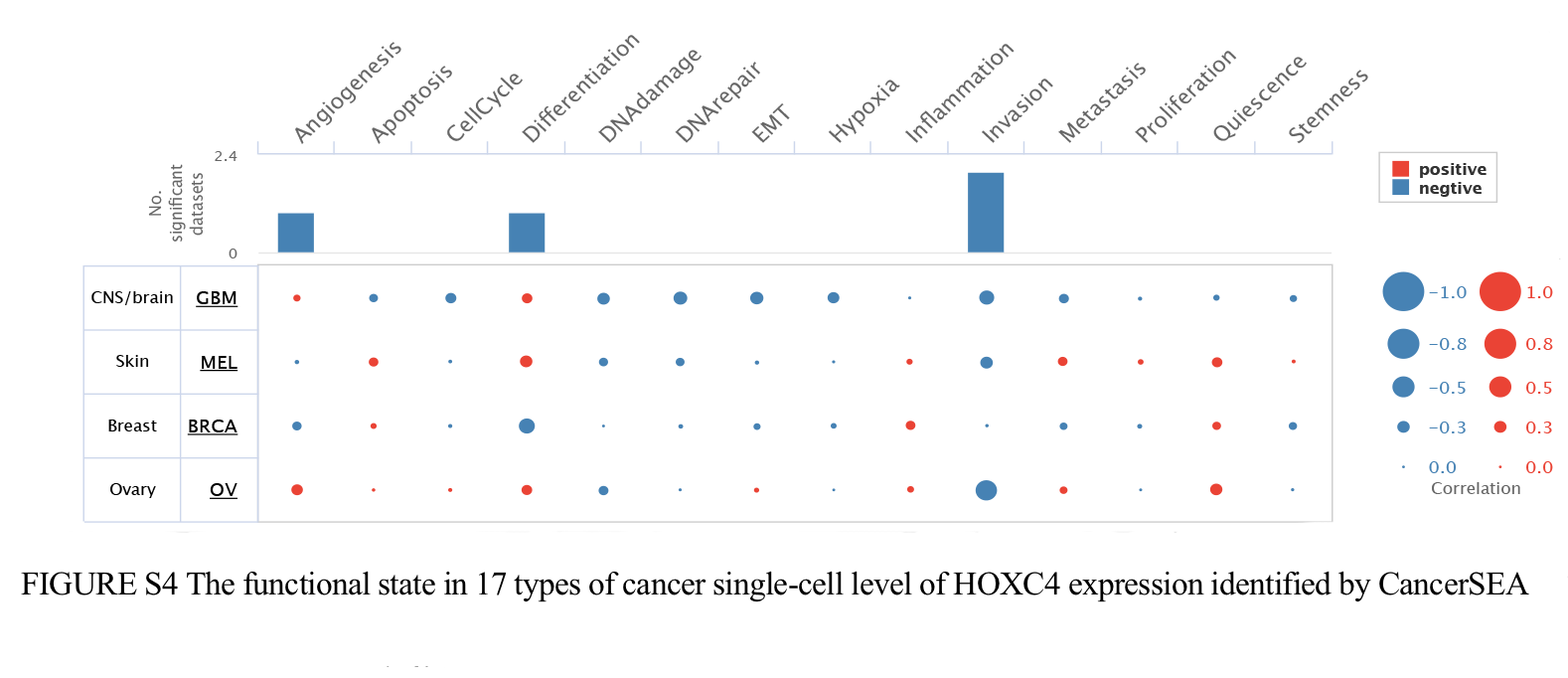

Supplement: Supplementary file 3 [file Image4.TIF]

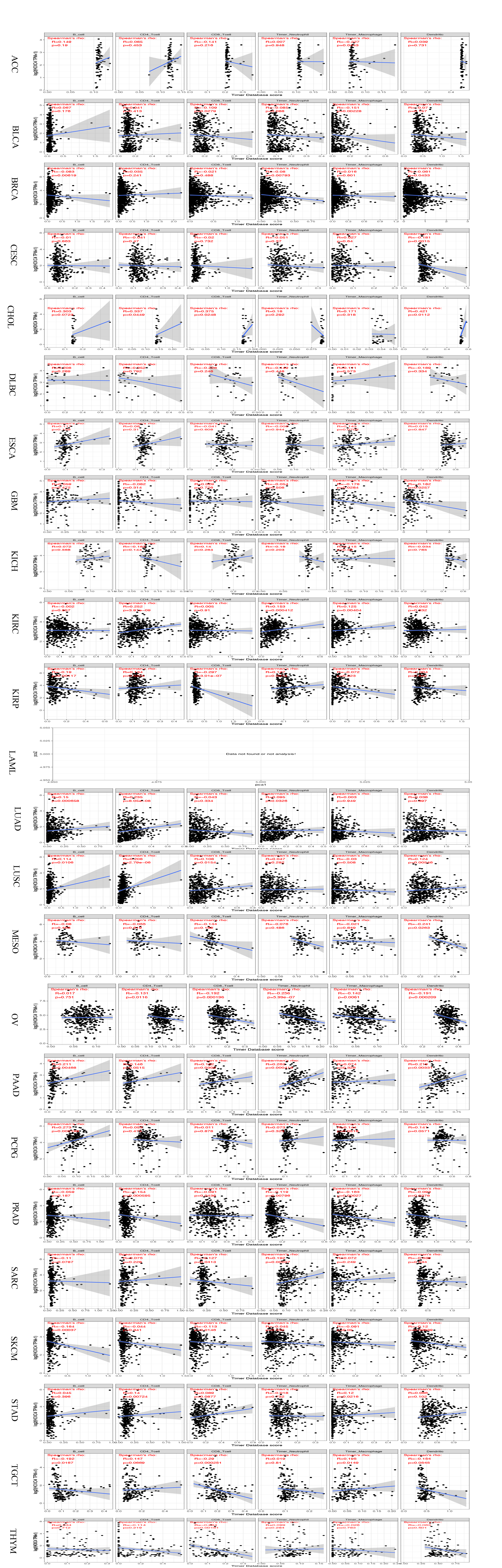

Supplement: Supplementary file 4 [file Image2.TIF]

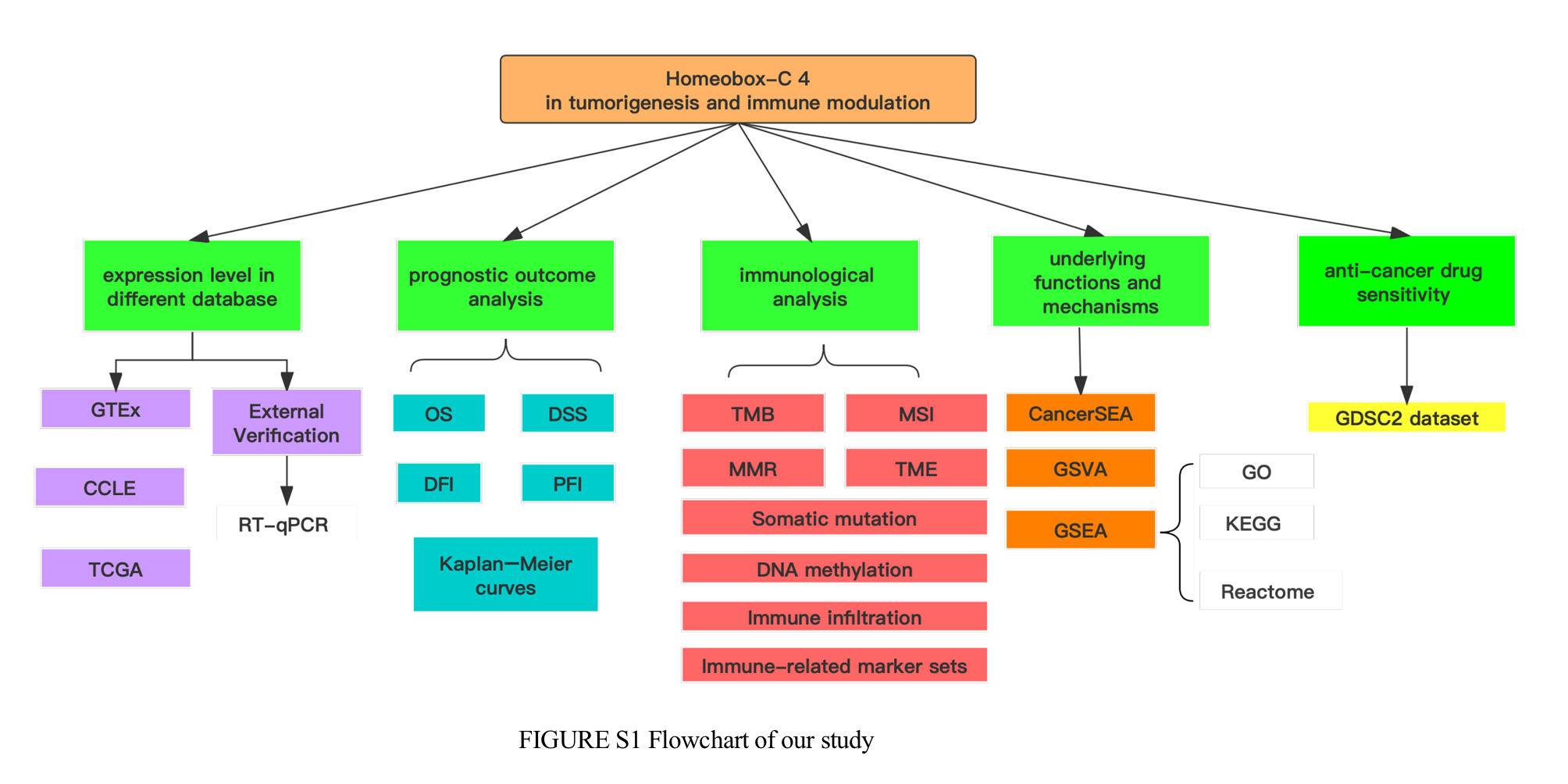

Supplement: Supplementary file 5 [file Image1.TIF]
